# The association between number of steps and the ambulatory blood pressure during leisure vs. work hours among cleaners

**DOI:** 10.1007/s00420-023-02015-1

**Published:** 2023-10-18

**Authors:** Vivian Rueskov Poulsen, Mathilde Baumann, Mette Korshøj

**Affiliations:** Department of Occupational and Social Medicine, Hospital Holbæk, Holbæk, Denmark

**Keywords:** Physical activity paradox, Cardiovascular disease, Cleaners, Ambulatory blood pressure, Walking

## Abstract

**Purpose:**

The physical activity paradox states occupational physical activity (OPA) to be hazardous and leisure time physical activity (LTPA) to be beneficial for health. Yet, the acute effects of OPA and LTPA on cardiovascular risk factors are sparsely investigated. The aim of this study was to investigate the acute effects on ambulatory blood pressure (ABP) from steps/hour during work and leisure time among cleaners.

**Methods:**

Data were obtained from a cluster randomized worksite intervention among 91 cleaners in Denmark and included a questionnaire, objective physical measurements, ABP (measured across 24 h), and steps/hour (measured during work and leisure time). A preliminary linear regression analysis was conducted as a mixed model including random intercept and slope, allowing for both within- and between-participant variability. We adjusted for sex, age, job seniority, medication use, smoking, self-reported fitness and BMI. Changes in ABP (mmHg) were estimated per 100 steps/hour.

**Results:**

The number of steps taken was not associated with ABP during either work or leisure. Moreover, the ABP did not seem to differ between exposure to steps taken during work (systolic − 0.42 mmHg, 95% Confidence Interval (CI): − 1.10–0.25, diastolic − 0.03 mmHg, 95% CI, − 0.45–0.39) and leisure time (systolic -0.47 mmHg, 95% CI, − 1.66–0.72, diastolic 0.25 mmHg, 95% CI, − 0.46–0.97).

**Conclusion:**

Our findings show no significant association between steps/hour and ABP and no contrasting effects between work and leisure time. These mechanisms fostering the divergent results need to be further investigated to improve the understanding of the physical activity paradox.

## Introduction

Previous literature suggests that occupational physical activity (OPA) is hazardous to health while leisure time physical activity (LTPA) has beneficial health effects (Coenen et al. 2018a, b; Holtermann et al. 2018; Li et al. 2013). For example, a review reports that a high level of OPA increases the risk of cardiovascular disease (CVD), whereas high levels of LTPA decrease the CVD risk (Li et al. 2013). These domain-specific differences in health effects of physical activity, known as the physical activity paradox, can partly be explained by higher heart rate during walking at work than during walking in leisure, perhaps indicating extra stimulus at work, which in turn, could contribute to the explanation of the negative effect on health (Coenen et al. 2018a, b). Yet, findings across studies investigating the physical activity paradox are inconsistent (Dalene et al. 2021).

An elevated intensity of OPA, measured as the relative aerobic workload (Karvonen et al. 1957), for several hours each working day, repetitive work, and prolonged static postures can lead to raised blood pressure (BP) (Gupta et al. 2020), and eventually hypertension (Clays et al. 2012). Hypertension is known as the leading preventable risk factor for cardiovascular disease, e.g. myocardial infarction and stroke, and all-cause mortality worldwide (Roth et al. 2018; Stanaway et al. 2018). One way to prevent hypertension is by increasing physical activity (Pedersen and Saltin 2006). As walking is a physical activity accessible to a large proportion of the population worldwide, as well as being independent of skill level and access to equipment (Oja et al. 2018; Saint-Maurice et al. 2020) and has minimal adverse effects (Morris and Hardman 1997), walking has the potential to prevent hypertension across individuals in different contexts.

The majority of research on the association between walking and health has focused on the number of steps *per day* omitting information on a domain, such as activity during work or leisure hours (Gupta et al. 2020). One study has investigated the effects of walking on BP in different domains, i.e. working and leisure time (Crowley et al. 2021). However, opposite to the physical activity paradox, this study finds a beneficial association between the number of steps, during work, and systolic BP, among blue-collar workers, and no associations among white-collar workers (Crowley et al. 2021). Hence, the mechanisms fostering such divergent effects in the different domains need to be further investigated.

Ambulatory BP (ABP) measurements have been found to be superior to conventional BP (CBP) measurements in predicting cardiovascular events (Hansen et al. 2007), and it has the advantage of possible measurements of BP in different domains (Clays et al. 2012). Thereby, ABP can be measured as an acute response to actual physical activity or body posture. Thus, ABP measurements offer an opportunity to measure BP without the biases of white-coat- and masked hypertension, leading to more accurate and non-biased measurements and diagnostics of patients with suspected hypertension (O’Brien et al. 2013). However, to our knowledge, no previous study has used this method to investigate the domain-specific effects of steps/hour. Furthermore, examining the acute impact of steps on ABP instead of separating measurements of steps and BP might lead to more precise results on the domain-specific effects of steps on ABP (Hansen et al. 2007). Therefore, the aim of this study was to investigate the association between time-synchronized ABP and the number of steps during work and leisure time among cleaners in Denmark.

## Methods

Data were obtained from baseline measures and subsequent technical diurnal measurements from a cluster randomized worksite intervention among cleaners. The study was approved by the Danish Data Protection Agency and the Ethics Committee for the regional capital in Denmark (journal number H-2–2011-116) and was conducted in accordance with the Helsinki Declaration. The study was registered as ISRCTN86682076 in the current controlled trials (2014) and is described in a protocol paper (Korshøj et al. 2012).

### Recruitment and participants

Cleaning companies in the suburban area of Copenhagen, Denmark were recruited by direct contact. All cleaning employees were invited to an information meeting where the project was described and assignment to voluntary participation was conducted via a screening questionnaire. A detailed recruitment procedure is described elsewhere (Korshøj et al. 2015). Participants were included if they were: employed as a cleaner ≥ 20 h/week, between 18 and 65 years of age, non-pregnant, and if they had signed an informed consent to participate in the study.

### Data collection

Data were collected at a baseline test and subsequently through technical diurnal measurements of ABP and steps/hour during work and leisure time within a maximum of four continuous days. The baseline test consisted of a structured interview, where information on sex, age, job seniority, medication use, smoking, and self-reported cardiorespiratory fitness were obtained. Moreover, the baseline test included objective physical measurements (body weight (Tanita BC418), height (seca model 213 1,721,009) and body mass index (BMI) = [body weight (kg)/body height (m2)] (Canoy 2008)), and BP measured on the left upper arm after 15 min of sitting at rest (Omron M6 comfort) (Korshøj et al. 2012). Technical diurnal measurements of ABP and steps/hour were processed in a custom-made software (Acti4) to synchronize time and domain (Skotte et al. 2014).

### Ambulatory blood pressure measurements

ABP measurements were performed with Spacelabs90217 (www.spacelabshealthcare.com) (Baumgart & Kamp 1998), by oscillometry, mounted on the non-dominant upper arm with a tube connecting the sampler to the cuff. The data sampler was mounted with elastic straps around the waist, and the frequency of measurements was every 20 min during waking hours and every 40 min during sleep (Clays et al. 2007, 2012). The participants were instructed to keep still and the arm at rest while the measurement was proceeding. If a measurement failed, the monitor automatically repeated the measure again a few minutes later. The participants were asked to wear the monitors 24 h/day on a day including work. They were instructed on how to treat the monitors and to remove the ABP device during showering. The 24-h recording was performed on the first or second day of the four days where steps/hour were measured. Furthermore, the 24-h recording was split into periods classified as work, leisure or sleep based on information from self-reported dairies where the participants were asked to write a log of working hours (when they started and ended work), sleeping time (got up in the morning and went to bed in the evening) and periods spent without monitors. ABP measurements were included when a minimum of 25% of measurements were complete (corresponding to the amount of measurements: five during work, eight during leisure and three during sleep), and all measures of ABP were visually checked and physiological outliers were excluded from analysis (systolic blood pressure < 80 and > 240 mmHg, diastolic blood pressure < 50 and > 130 mmHg) (Korshøj et al. 2016).

### Measurements of the number of steps

The number of steps was sampled by Actigraph GT3X + , a triaxial accelerometer, with a dynamic range of ± 6 G, sampled with the precision of 12 bit. The Acti4 software was used to process raw data and estimate time spent in different body positions and activities (Skotte et al. 2014; Stemland et al. 2015). The accelerometers were initialized for recording and data were downloaded using the manufacturer’s software (ActiLife version 5.5). Actigraphs were mounted on the skin with adhesive tape on the right thigh at the most muscular part of the quadriceps femoris, medial to the front of the iliac crest and the top of the patella, orientated with the x-axis pointing downwards, y-axis horizontally to the left and z-axis horizontally forward (Skotte et al. 2014). The Actigraph signals were sampled at 30 Hz to derive the number of steps.

### Assessments of potential confounders

The selection of covariates (potential confounders) was based on prior related studies and findings in the literature (Coenen et al. 2018a, b; Coenen et al. 2018a, b; Kjeldsen 2018; Kodama 2009; Merellano-Navarro et al. 2017; Reckelhoff 2001), e.g. associations between BP and age have previously been found (Kjeldsen 2018), and other findings show age to associate with physical fitness performance, such as walking (Merellano-Navarro et al. 2017). Furthermore, sex differences in BP (Reckelhoff 2001) as well as walking (Allen and Vella 2015; Pollard and Wagnild 2017) have been observed. Additionally, it is well-established that drug therapy is associated with BP control (Williams et al. 2018). Also, smoking has been associated with BP (Virdis et al. 2010) as well as walking (Allen and Vella 2015). Cardiorespiratory fitness has previously been associated with cardiovascular events (Kodama 2009) and BP (Cornelissen and Smart 2013). Previous findings, however, also indicate that cardiorespiratory fitness moderates the association between OPA and ischemic heart disease (Holtermann et al. 2010). Therefore, cardiorespiratory fitness was included first as a confounder and later as a moderator in our analyses.

Participants were asked to indicate their sex, date of birth, for estimation of age, and the number of years they had had their current job type, for estimation of job seniority. In this study, participants were asked if they within the past four weeks had used any medication prescribed by a doctor, i.e. cholesterol-lowering drugs, diuretics, antihypertensives, antidepressants, pain killers and/or others (response options: yes or no). Participants were asked: “Do you smoke?” (response options: 1) yes, on a daily basis; 2) yes, sometimes; 3) I have previously smoked, but not anymore and 4) I have never smoked). Smoking responses were dichotomized into yes; yes, sometimes and no, never, previously. To measure self-reported cardiorespiratory fitness, the participants were asked: “How would you perceive your fitness compared to other people in your age group and the same sex as you?” The participants could range their level on a scale from 1 to 10; with 1 representing *poor* and 10 representing *good* (Strøyer et al. 2007)*.* Fitness was also objectively measured by a step test (Aadahl et al. 2013). However, self-reported cardiorespiratory fitness was chosen over objectively measured cardiorespiratory fitness to ensure a higher number of participants to be included in the analysis. To test if self-reported cardiorespiratory fitness corresponded to the actual level of the participants’ objectively measured cardiorespiratory fitness, the normal distribution curves of each variable were assessed and concluded to be comparable.

## Statistical analysis

IBM SPSS software version 27.0 was used for all statistical analyses. The distribution of step data and ABP were checked and considered normally distributed. The remaining continuous variables (age, BMI, job seniority, self-reported cardiorespiratory fitness) were likewise checked and considered normally distributed.

Descriptive analyses of baseline characteristics were performed (Table 1). The number of participants and percentage distribution were reported for categorical variables and means, and Standard Division (SD) were calculated for the continuous variables.

**Table 1:** Description of the study population including distribution of participants across categorical variables and mean ± SD for continuous variables

	*n* (%)	Mean ± SD
Number of participants	91 (100.0)	
Sex		
Men	22 (24.2)	
Women (ref)	69 (75.8)	
Prescribed medicines		
Yes	12 (13.2)	
No (ref)	79 (86.8)	
Smoking		
Yes; Yes, sometimes	23 (25.3)	
No; never; previously (ref)	62 (68.1)	
Age (years)	91	45.4 ± 8.2
BMI (kg/m^2^)	91	27.0 ± 4.6
Job seniority (years)	88	12.5 ± 7.7
Self-reported cardiorespiratory fitness	90	5.1 ± 1.96
Steps/hour at work	90	1332.6 ± 404.1
Steps/hour in leisure	90	526.0 ± 234.1
Systolic baseline conventional blood pressure (mmHg)	91	123.3 ± 20.8
Ambulatory blood pressure (mmHg) at work	84	123.1 ± 14.2
Ambulatory blood pressure (mmHg) in leisure	91	121.8 ± 13.3
Diastolic baseline conventional blood pressure (mmHg)	91	83.1 ± 12.1
Ambulatory blood pressure (mmHg) at work	84	80.9 ± 8.3
Ambulatory blood pressure (mmHg) in leisure	91	77.7 ± 8.2

Description of study population (*n* = 91) at baseline measurements, mean ± SD or *n* (%)

As we included repeated measurements of the (continuous) dependent outcome variables for all participants, a linear regression analysis was conducted as a mixed model including random intercept and random slope, allowing for both within-participant and between-participant variability. For exposure variables (steps/hour) we calculated the mean values for work hours and leisure time, respectively. Changes in ABP (mmHg) were estimated as an increase/decrease in APB per 100 steps/hour. Independent exposure variables were included as continuous variables in all analyses. Missing measurements were not imputed (Twisk et al. 2013). To avoid unnecessary adjustment, covariates were included stepwise in the analyses. First, we conducted a raw model (Model 1, Table 2) with no adjustments. Second, we adjusted for demographic characteristics, i.e. sex, age, and job seniority (Model 2, Table 2). Next, we adjusted for behavioral factors, i.e. use of medicine, smoking habits, self-reported cardiorespiratory fitness, and BMI (Model 3, Table 2).

**Table 2:** The association between mean steps/hour and ambulatory blood pressure (ABP) (mmHg) in working hours and leisure time. Estimates show the change in ABP pr. 100 steps increase/hour

ABP (mmHg)	Working hours					Leisure time				
Model 1*	Estimate	SE	95% CI	*p*	*n*	Estimate	SE	95% CI	*p*	*n*
Systolic	– 0.02	0.36	– 0.73–0.68	0.95	90	– 0.75	0.61	– 1.97–0.47	0.22	90
Diastolic	0.23	0.21	– 0.19–0.65	0.29	90	0.09	0.36	– 0.64–0.82	0.81	90
Model 2**										
Systolic	– 0.28	0.34	– 0.96–0.41	0.43	87	– 0.52	0.60	– 1.71–0.66	0.38	87
Diastolic	– 0.06	0.21	– 0.35–0.47	0.77	87	0.20	0.35	– 0.50–0.91	0.56	87
Model 3***										
Systolic	– 0.42	0.34	– 1.10–0.25	0.21	81	– 0.47	0.60	– 1.66–0.72	0.43	81
Diastolic	– 0.03	0.21	– 0.45–0.39	0.90	81	0.25	0.36	– 0.46–0.97	0.48	81

*No adjustments

**Adjusted for sex, age, and job seniority

***Model 2 + adjustment for medication use, smoking, self-reported cardiorespiratory fitness and BMI

In the sensitivity analyses, we repeated Model 3 with the inclusion of a multiplicative interaction term (steps/hour x self-reported cardiorespiratory fitness) to capture the potential moderating effect of cardiorespiratory fitness on the association between steps/hour and ABP during working hours and leisure time, respectively.

## Results

### Study population and baseline characteristics

**Fig. 1:**
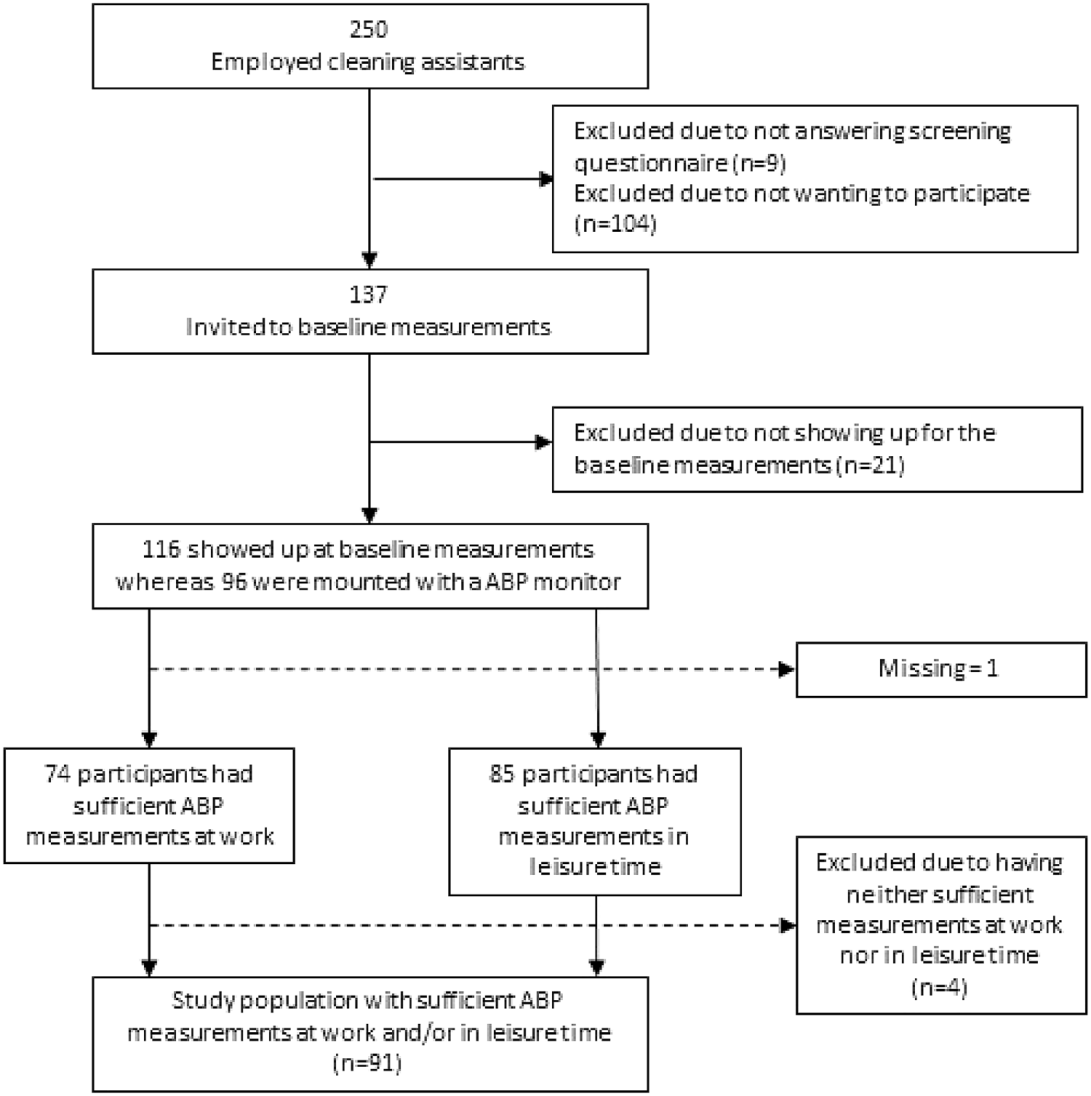
Flowchart of inclusion and exclusion of participants in the study on the association between steps/hour and ambulatory blood pressure (ABP) among cleaners in Denmark during working hours and leisure time, respectively

All of the three contacted companies in the suburban area of Copenhagen, Denmark, agreed to participate. The study was presented to 250 cleaning assistants at these companies. Of those, 137 (45%) agreed to participate and 116 underwent the baseline measurements. In total, 96 participants had an ABP monitor mounted (23 male and 73 female), and 91 participants had a sufficient number of ABP measurements to be included in the statistical analysis (Fig. 1).

In total, 75.8% of participants were women, 13.2% used medication and 25.3% smoked (Table 1). The mean age for all participants was 45.4 years (SD ± 8.2), the mean BMI was 27.0 kg/m^2^ (SD ± 4.6), mean job seniority was 12.2 years (SD ± 7.7), and participants on average perceived their cardiorespiratory fitness level as moderate (mean = 5.1, SD ± 1.96) compared to peers. Mean steps/hour were 1332.6 (SD ± 404.1) during work and 526.0 (SD ± 234.1) during leisure time. The systolic BP was 123.3 mmHg (SD ± 20.8) and the mean diastolic BP was 83.1 mmHg (SD ± 12.1) at baseline (Table 1).

### The effect of steps/hour on ABP at work and in leisure time

The analyses showed no associations between ABP and steps/hour in work and leisure time. The ABP did not seem to differ by the domain of the exposure to steps; during work (systolic − 0.42 mmHg/100 steps/hour, 95% CI: − 1.10–0.25, diastolic -0.03 mmHg/100 steps/hour, 95% CI, − 0.45–0.39) and leisure (systolic -0.47 mmHg/100 steps/hour, 95% CI, − 1.66–0.72, diastolic 0.25 mmHg/100 steps/hour, 95% CI, − 0.46–0.97) (Model 3, Table 2).

### Sensitivity analysis

The sensitivity analyses did not show self-reported cardiorespiratory fitness to moderate the association between steps/hours during work hours and the systolic or diastolic ABP. Neither did fitness level moderate the association between steps/hours in leisure time and the diastolic ABP. However, we cannot rule out the possibility that cardiorespiratory fitness moderates the association between steps/hour in leisure time and the systolic ABP on an 80% significance level (*p* = 0.132) (Krause et al. 2017). A complementary analysis stratified by cardiorespiratory fitness level above/below the median (i.e. 5.00) was therefore conducted. However, when stratified on fitness level, we found no statistically significant changes in the estimate, thus, these analyses did not change our overall conclusion and are therefore not presented in detail.

## Discussion

### Comparison with previous findings

The literature describing the associations between physical activity and CVD is inconsistent, and it has been convincingly argued that more research that differentiates between the role of physical activity during work vs. leisure time for CVD is needed (Krause 2010). Moreover, there is evidence that daytime ABP monitoring is superior to CBP measurement in the prediction of CVD, however, studies that include such ABP measurements are lacking (Hansen et al. 2007). This study examined the association between the domain-specific number of steps/hour and time-synchronized ABP among 91 cleaners in Denmark. Thus, to our knowledge, this study is the first one investigating the acute effect of steps on BP, and a direct comparison with previous studies may not be possible.

The main findings of this study are that there were no significant effects of the number of steps on the ABP as well as no contrasting effects between work and leisure time in the associations of ABP and steps/hour. Thus, we did not find any adverse health effects of OPA, nor any beneficial health effect of LTPA, which is distinct from the physical activity paradox advocating that LTPA promotes health, while high OPA impairs health (Gupta et al. 2020; Holtermann et al. 2012). Previously, Johansson et al. (2022) reported that reallocating time from being sedentary to walking, during leisure, was beneficial for BP, whereas during work, it was harmful for BP. Also, the results from our study could not support the previously shown association between steps/hour during work and a lowered CBP (Crowley et al. 2021) during neither work nor leisure time. Additionally, our findings are opposite to a number of previous studies showing harmful effects of self-reported OPA, such as lifting and carrying loads, on the CBP (Allesøe et al. 2016; Virkkunen et al. 2007; Åstrand et al. 2003), as well as the ABP (Clays et al. 2012). But, similarly to our results, Clays et al. (2012) found no associations between objectively measured OPA and ABP. Holtermann et al. (2018) introduced six reasons for the physical activity paradox; one being that the intensity of OPA does not reach a level where individuals will gain cardiorespiratory fitness. Other explanations for the effects of OPA included insufficient time for recovery, long durations, and static and constrained postures and activities (Holtermann et al. 2018). Considering the contrasting results regarding the effects of OPA on BP with respect to the work of Holtermann et al. (2018), one explanation for not finding an association between steps/hour and ABP could be that different types of OPA, such as steps vs. heavy lifting, have different effects on the cardiovascular system and thus the BP.

Our study did not find a significant association between the number of steps at leisure and ABP, i.e. ABP did not decrease significantly with a greater number of steps taken during LTPA. In the same way, the study by Crowley et al. (2021) showed no statistically significant association between the number of steps during leisure and systolic BP (Crowley et al. 2021). These results are in contrast to previous findings of LTPA having a protective effect on health (Clays et al. 2012; Saint-Maurice et al. 2019) and being associated with a reduced risk of CVD (Holtermann et al. 2021). More research on the domain-specific acute effects of different physical activities, in particular steps/hour, would shed light on this.

The combination of high physical work demands and low cardiorespiratory fitness has been associated with increased risk for cardiovascular disease mortality (Holtermann et al. 2021). This is important knowledge considering the fact that cleaners often experience high physical work demands (Korshøj et al. 2012), and therefore are at risk of overload or damage to the cardiovascular system (Holtermann et al. 2009). In this study, we could not rule out the possibility of cardiorespiratory fitness level to moderate the association between steps/hour during leisure time and the systolic ABP, which somehow indicates that fitness level may protect against or worsen the effects of OPA on risk factors for CVD, such as increased ABP. However, when stratified on cardiorespiratory fitness level, we found no statistically significant results. Thus, whether the number of steps during work hours is beneficial or harmful to cleaners’ cardiovascular health and whether the cardiorespiratory fitness level moderates the effect of steps, cannot be concluded from this study.

### Strengths and limitations

This study is strengthened by the use of objective measurements of steps/hour, ABP, and BMI among cleaners, which decreases the risk of subjective recall bias. Furthermore, this study was strengthened by technical diurnal measurements ABP measurements, previously found to be a stronger predictor for cardiovascular disease than CBP measurements (Hansen et al. 2007). ABP measurements that are time-synchronized to step counts, seem to be a reliable method to measure the acute effect of steps/hour on the ABP. However, cleaning also involves several physically demanding tasks that increase the risk of high BP (Korshøj et al. 2012). Thus, the associations we investigated in this study might have been biased by the exposure of other risk factors for high BP, such as high relative aerobic workload (Korshøj et al. 2015).

To minimize the risk of bias the analyses were adjusted for several confounders, however, we cannot preclude that our findings may have been affected by unmeasured confounding. Moreover, a limitation of this study may be, that we chose to adjust the analyses for self-reported cardiorespiratory fitness level and not objectively measured fitness, to include more participants in the analyses. The drawback of this is that we may have increased the risk of recall bias on this specific topic. However, by comparing the normal distribution curves for objective and self-reported cardiorespiratory fitness we found that the two measurements were comparable. Additionally, self-reported fitness has previously been identified as a strong independent predictor of risk factors of CVD and all-cause mortality (Holtermann et al. 2015). Thus, it is likely that self-reported fitness did not introduce a bias in our study.

## Conclusion

In conclusion, this study among cleaners in Denmark demonstrated no significant association between diurnal time-synchronized steps/hour and ABP, and no contrasting findings of ABP when exposed to walking during work and leisure time. All in all, this study underlines the need to investigate the acute mechanisms contributing to divergent results in studies on the physical activity paradox. This is important to be able to identify and develop preventive initiatives in the future for those workers who actually experience harmful effects from OPA on their cardiovascular health.

## Data Availability

The data that support the findings of this study are available from Mette Korshøj on reasonable request.

## Abbreviations

ABP: Ambulatory blood pressure
BMI: Body mass index
BP: Blood pressure
CBP: Conventional blood pressure
CI: Confidence interval
CVD: Cardiovascular disease
LTPA: Leisure time physical activity
OPA: Occupational physical activity
SD: Standard division
